# Revisional Procedures after Sleeve Gastrectomy for Weight Recurrence or Inadequate Weight Loss: An Analysis of the MBSAQIP Database

**DOI:** 10.3390/jcm12185975

**Published:** 2023-09-15

**Authors:** Karl Hage, Juan S. Barajas-Gamboa, Gustavo Romero-Velez, Matthew Allemang, Salvador Navarrete, Ricard Corcelles, John Rodriguez, Omar M. Ghanem, Matthew Kroh, Jerry T. Dang

**Affiliations:** 1 Department of Surgery, Mayo Clinic, Rochester, MN 55905, USA; 2 Digestive Disease Institute, Cleveland Clinic Abu Dhabi, Abu Dhabi P.O. Box 112412, United Arab Emirates; 3 Digestive Disease Institute, Cleveland Clinic, Cleveland, OH 44195, USA

**Keywords:** sleeve gastrectomy, gastric bypass, duodenal switch, bariatric conversions, conversion safety

## Abstract

Introduction: The safety of conversional bariatric procedures after sleeve gastrectomy (SG) for weight recurrence (WR) or inadequate weight loss (IWL) is debated due to limited evidence. Conversion options include Roux-en-Y gastric bypass (RYGB), single anastomosis duodeno-ileal bypass (SADI), and biliopancreatic diversion with duodenal switch (BPD-DS). We aimed to compare serious complications and mortality rates between these procedures within 30 days. Methods: Using the 2020 and 2021 MBSAQIP databases, we identified patients who underwent a conversion from SG to RYGB, SADI, or BPD-DS. We performed a multivariable logistic regression to assess predictors of 30-day complications and mortality. Results: Among 7388 patients (77.6% RYGB, 8.7% SADI, 13.7% BPD-DS), those undergoing SADI and BPD-DS had higher preoperative body mass index. Conversion reasons included WR (63.0%) and IWL (37.0%). SADI and BPD-DS patients had longer operative times (*p* < 0.001) and higher leak rates (*p* = 0.001). Serious complications, reoperations, readmissions, and 30-day mortality were similar across groups. Conversion procedure type was not an independent predictor of complications. Conclusion: RYGB was the most performed conversional procedure after SG. The study indicated a similar safety profile for revisional RYGB, SADI, and BPD-DS, with comparable 30-day complications and mortality rates. However, SADI and BPD-DS patients had longer operative time and higher leak rates.

## 1. Introduction

Obesity is a rapidly growing epidemic, affecting over 650 million people worldwide [1]. Metabolic and bariatric surgery (MBS) remains one of the most effective treatments for obesity, with sleeve gastrectomy (SG) being the most commonly performed bariatric procedure worldwide [2]. However, long-term weight loss outcomes after SG are not always optimal, and some patients seek revisional surgery. The rates of revisional procedures after SG were reported to reach up to 12.2% at 10 years’ follow-up [3]. Although Roux-en-Y gastric bypass (RYGB) is the preferred procedure for addressing gastroesophageal reflux disease (GERD) and other postoperative complications after SG [4], there remains controversy regarding the ideal procedure for patients presenting with weight recurrence (WR) or inadequate weight loss (IWL) after SG [5].

The most commonly performed revisional bariatric procedures for these cases include RYGB, single-anastomosis duodeno-ileal bypass (SADI), and biliopancreatic diversion with duodenal switch (BPD-DS) [6,7]. Lee et al. conducted a systematic review of SG conversions and found that conversions to RYGB, SADI, and BPD-DS were equally safe. However, despite including six studies, this review only had a total of 377 patients [8], which justifies the need for a larger nationwide study to validate these outcomes.

Given the limitations of previous studies and the growing need to evaluate the revisional surgery after SG, our study aims to determine the rate of 30-day serious complications and mortality of conversions due to IWL/WR from SG to RYGB, SG to SADI, and SG to BPD-DS using new revisional variables that were recently added to the Metabolic and Bariatric Surgery Accreditation and Quality Improvement Program (MBSAQIP) nationwide database.

## 2. Materials and Methods

### 2.1. Data Source

A retrospective analysis of the MBSAQIP data registry was conducted, focusing on 2020 and 2021 due to the inclusion of additional variables on revisional surgery that had not been previously reported. The MBSAQIP collects clinical data from 902 accredited centers in the United States and Canada. This registry prospectively captures standardized data on pre-, intra-, and postoperative variables specific to bariatric surgery patients. This study included patients who underwent a previous SG with subsequent conversion to RYGB, SADI, or BPD-DS due to WR or IWL. Patients who had non-weight-related indications for conversion, such as GERD, dysphagia, or other, were excluded. Patients who had a revisional bariatric surgery following any primary procedure other than SG, as well as open and endoscopic procedures, were also excluded.

### 2.2. Variables

Basic demographic data including age, sex, and race, were collected. Comorbidities included the following: type 2 diabetes mellitus (T2DM), hypertension, GERD, chronic obstructive pulmonary disease (COPD), hyperlipidemia, chronic steroid use, chronic kidney disease, dialysis dependency, venous stasis, preoperative therapeutic anticoagulant use, and obstructive sleep apnea. Patient history included smoking status, previous venous thromboembolism (VTE), myocardial infarction (MI), percutaneous coronary intervention, and major cardiac surgery. Functional status and American Society of Anesthesiologists (ASA) Physical Status classification were included. Perioperative variables included conversion procedure performed, operative time, robotic assistance, concurrent paraesophageal hernia repair (identified using CPT codes 39599, 43280, 43327, 43281, 43282, 43332, 43333, 43336, 43337), concurrent lysis of adhesions (CPT codes 44005, 58660), and length of hospital stay.

As defined in the MBSAQIP manual, preoperative GERD is based on the presence of symptoms in addition to the regular use of over-the-counter medication within 30 days of the revisional procedure. For weight-related indications such as IWL and WR, the MBSAQIP does not provide specific definitions of these variables. Both variables are included as the ‘’final indication’’ variable for revisional surgery. The MBSAQIP aims to capture the indication for revisional surgery without clarifying the exact definitions, as these can vary widely by institution.

### 2.3. Objectives

The primary objective of this study was to determine the rate of major complications and mortality of conversions from SG to RYGB, SADI, and BPD-DS. Secondary objectives included identifying and characterizing indications for conversions to RYGB, SADI, and BPD-DS. We defined patients who experienced major or serious complications based on the Clavien-Dindo classification for postoperative complications. Patients who presented with at least one of the following complications were considered to have a grade II Clavien-Dindo complication or higher: urinary tract infection, pneumonia, venous thrombosis, need for transfusion, deep incisional surgical site infection, pulmonary embolism, acute renal failure, cardiac arrest, myocardial infarct, progressive renal failure, need for mechanical ventilation, sepsis, septic shock, unplanned ICU admission, and death.

### 2.4. Statistical Analysis

Categorical data were expressed as percentages, and continuous data as weighted mean ± standard deviation (SD). Univariate analyses were used to determine baseline differences between groups, using chi-squared tests for categorical data and independent sample t-tests for continuous data. Univariate logistic regression was used to compare differences between patients in the RYGB, SADI, and BPD-DS cohorts.

Multivariable logistic regression analysis was used to identify predictive factors for serious complications and mortality within 30 days. The available case method addressed missing data as all variables had less than 5% missing. Patients’ baseline demographics and operative factors were included in the model. Any variable with a *p*-value < 0.05 in univariate analysis was included in multivariable analysis. Variables were checked for collinearity via the variable inflation factors method. The area under the receiver operating characteristic (AUROC) curve and Brier score were used to assess validity and calibration of the multivariable model. Statistical analysis was performed using Stata 17 [9].

## 3. Results

### 3.1. Participants

**Figure 1:**
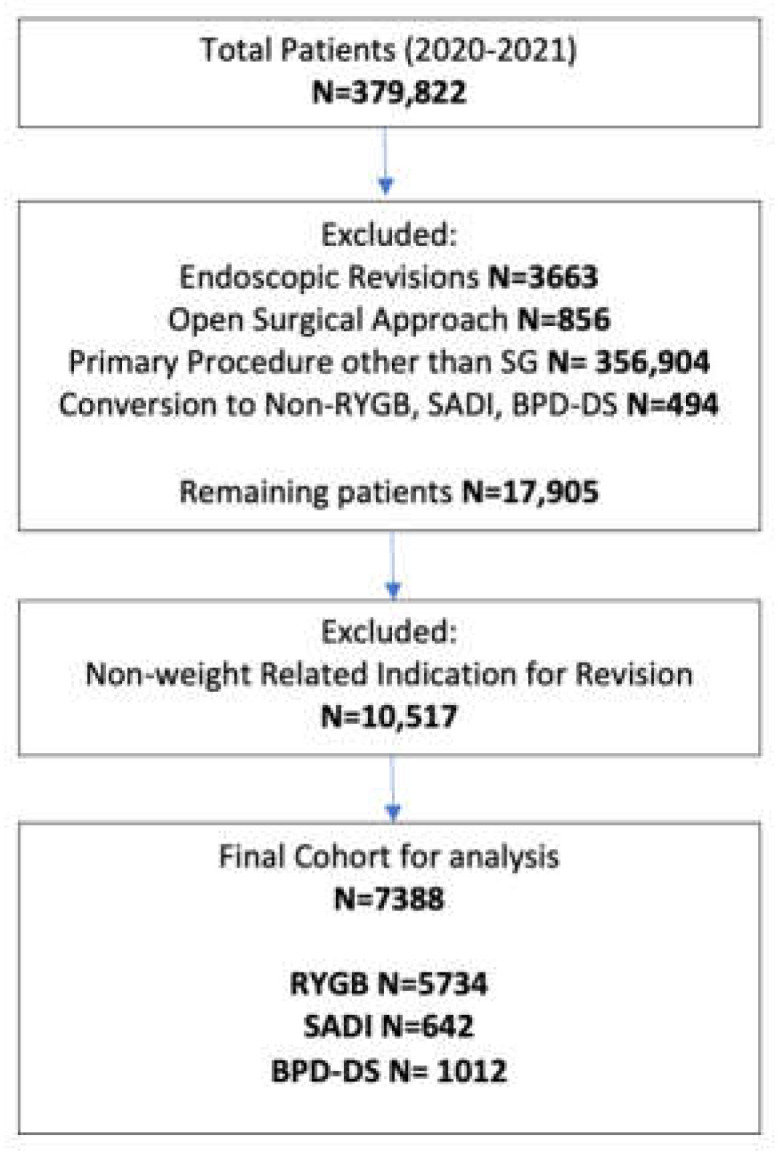
Patient flow chart. SG, primary sleeve gastrectomy; RYGB, conversion from sleeve gastrectomy to Roux-en-Y gastric bypass; SADI, conversion from sleeve gastrectomy to single-anastomosis duodeno-ileal bypass; BPD-DS, conversion from sleeve gastrectomy to biliopancreatic diversion with duodenal switch.

A total of 379,822 adult patients undergoing revisional bariatric surgery were identified in the 2020–2021 MBSAQIP databases. Out of these patients, 3663 were excluded as they had endoscopic revisional surgery, and another 856 patients were excluded for having an open surgical approach. We also excluded patients who had a revisional bariatric surgery following any primary procedure other than sleeve gastrectomy (SG) (n = 356,904). Additionally, patients who had a conversion from SG to any procedure other than RYGB, SADI, or BPD-DS were also excluded from the analysis (n = 494). This resulted in 17,905 patients who underwent a conversion from SG to either RYGB, SADI, or BPD-DS. Furthermore, the patients were stratified by indication for conversion and patients who had a non-weight-related indication such as GERD (n = 9093), dysphagia (n = 262), persistent comorbidities (n = 152), or other gastrointestinal complications (n = 1010) were excluded. In our final cohort, 7388 patients (86.7% female; age 44.9 ± 10.2 years; mean pre-operative BMI 43.8 ± 7.6 kg/m^2^) were analyzed (Figure 1). Patients’ baseline demographics and obesity-related medical conditions are presented in Table 1.

**Figure 2:**
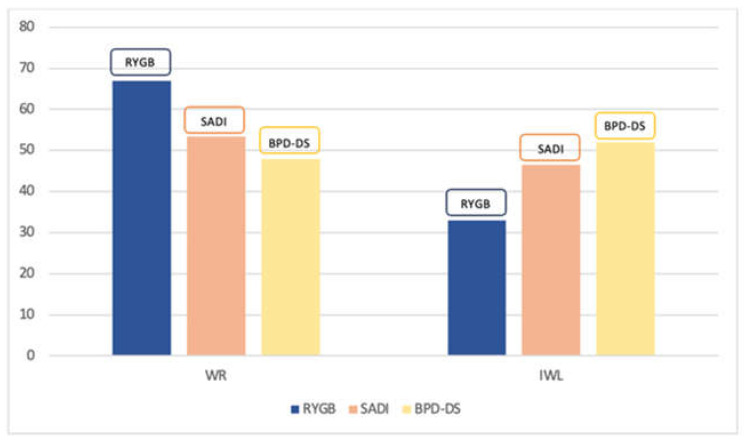
Indication for revisional surgery after sleeve gastrectomy. WR: weight recurrence; IWL: inadequate weight loss; RYGB: Roux-en-Y gastric bypass; SADI: single anastomosis duodeno-ileal bypass; BPD-DS: biliopancreatic diversion with duodenal switch.

RYGB was the most performed revisional surgery after primary SG in our cohort (n = 5734; 77.6%) followed by BPD-DS (n = 1012; 13.7%) and SADI (n = 642; 8.7%). Analysis of specific conversion indications for our cohort (after exclusion of non-weight related indications) showed that WR was the primary reason for conversion (n = 4658; 63%) overall, followed by IWL (n = 2730; 37%). When separating by procedure type, WR was also the main conversion indication for SG to RYGB and for SG to SADI conversions. However, patients undergoing conversion from SG to BPD-DS had IWL as the most common conversion indication (52% vs. 48%) (Figure 2).

Analysis of patient baseline characteristics and demographics demonstrated that there was no statistical difference in age at procedure or race between the three groups; however, patients who converted to RYGB were more likely to be female compared to the SADI and BPD-DS groups, respectively (87.5% vs. 83.6% and 83.7%; *p* < 0.001). Additionally, the BPD-DS cohort had a statistically higher mean preoperative BMI (46.4 kg/m^2^) than the SADI (45.5 kg/m^2^) and RYGB (43.2 kg/m^2^) groups, respectively (*p* < 0.001). Lastly, the BPD-DS group also had the highest proportion of patients classified as ASA class 4–5 (7.3%) compared to the SADI (4.7%) and RYGB (2.7%) groups (*p* < 0.001) (Table 1).

### 3.2. Baseline Comorbidities

In our cohort, GERD was the most prevalent preoperative obesity-related medical condition (n = 3406; 46.1%). Hypertension (n = 2774; 37.5%), obstructive sleep apnea (n = 2074; 28.1%), dyslipidemia (n = 1312; 17.8%), and diabetes (n = 1011; 13.7%) were also commonly found. Comparison of baseline comorbidities between the three revisional procedures demonstrated that SADI and BPD-DS cohorts had higher rates of diabetes (SADI, 16.6% BPD-DS, 15.5% RYGB, 13.1%; *p* = 0.015) and sleep apnea (SADI, 31.5% BPD-DS, 31.8% RYGB, 27%; *p* = 0.001) while RYGB had a higher rate of preoperative GERD (SADI, 36.5% BPD-DS, 35% RYGB, 49.2%; *p* < 0.001). In addition, patients who converted to SADI were more likely to have a history of chronic kidney disease (SADI, 0.9% BPD-DS, 0.2% RYGB, 0.3%; *p* = 0.012) and venous thromboembolism (SADI, 5.8% BPD-DS, 3.9% RYGB, 2.6%; *p* < 0.001) (Table 1).

### 3.3. Peri- and Postoperative Outcomes

Patients undergoing SADI or BPD-DS had a marginally longer operative time compared to the RYGB cohort. The mean operative time was 149.1 ± 67.8 min and 148.8 ± 63.0 min for the SADI and BPD-DS groups, respectively, compared to 141.0 ± 66.1 min for patients undergoing RYGB (*p* < 0.001). Additionally, the robotic-assisted surgery rate was higher in the BPD-DS (n = 353; 34.9%) and SADI (n = 194; 30.2%) groups compared to the RYGB cohort (n = 1233; 21.5%) (*p* < 0.001). However, conversion to RYGB was associated with higher rates of concurrent paraesophageal hernia repair (24.2% vs. 15.3% and 14.3%) than SADI and BPD-DS (*p* < 0.001). Apart from higher rates of anastomotic leak (1.6% SADI, 1.3% BPD-DS, 0.5% RYGB; *p* = 0.001) and deep surgical site infections (1.6% SADI, 1.8% BPD-DS, 1.0% RYGB; *p* = 0.049), the SADI and BPD-DS groups presented similar rates of serious complications compared to RYGB. There were no statistical differences in reoperation and readmission and mortality rates within 30 days between the three cohorts. Lastly, it is also important to highlight the very low mortality rate associated with all three of these procedures (0% SADI, 0.1% BPD-DS, 0.1% RYGB; *p* = 0.715) after 30 days of revisional surgery (Table 2).

Using a multivariable logistic regression model, we were able to identify certain parameters predictive of serious complications after revisional bariatric surgery. A longer operative time (per hour) [adjusted odds ratio 1.18; 95% CI 1.09–1.28], as well as black race [adjusted odds ratio 1.32; 95% CI 1.07–1.28], were significantly associated with higher rates of serious complications (*p* < 0.001 and *p* = 0.01, respectively). On the other hand, conversional procedure type and sex were not independently predictive of serious complications on multivariable analysis (Table 3).

## 4. Discussion

The increasing prevalence of conversional bariatric surgery after primary SG has encouraged us to examine the 2020–2021 MBASQIP to determine the safety of conversions from SG to RYGB, SADI, or BPD-DS due to weight-related causes. Our findings indicate that RYGB is still the most commonly performed revisional surgery after SG (77.6%) and that GERD is the most prevalent comorbidity found in this specific cohort of patients (46.1%). Additionally, despite having longer operative times, and higher rates of anastomotic leak and surgical site infections, conversion to both SADI and BPD-DS presented a similar safety profile compared to RYGB within 30 days. Indeed, reoperations, readmissions, and mortality were similar between the three cohorts. Finally, we demonstrated that conversional procedure type was not independently predictive of serious complications after revisional surgery.

The published literature has identified several indications for revisional surgery after SG with the most common causes being GERD and weight-related issues [10]. There is still ongoing debate regarding the leading cause for conversions, with some studies identifying WR and IWL as the most prevalent indications [11]. There is a general consensus that supports RYGB as the optimal revisional procedure after SG to treat GERD given the anti-reflux mechanism it confers [12,13]. Despite a recent study demonstrating that 24.4% of patients develop GERD at medium-term follow-up after RYGB, this procedure is still considered the ideal revisional surgery for patients with concurrent WR/IWL and GERD [14]. Nevertheless, data describing the ideal procedure for weight-related complications after SG remain scarce. In an attempt to evaluate SG conversion outcomes, Clapp et al. performed a recent analysis of the MBSAQIP national database and identified that RYGB was the most common conversional procedure after SG and that there were no differences in weight outcomes, reoperation, reintervention, and readmission rates between conversions to RYGB, SADI, and BPD-DS [15]. Nevertheless, this study only included data from the 2020 MBASQIP database and evaluated all the indications for conversions (i.e., weight-related and non-weight related). In our study, we included the 2020 and 2021 databases and limited the analysis to weight-related indications for conversions only, thus increasing homogeneity in our comparison.

The safety profile of revisional bariatric surgery remains a main concern for patients with weight gain after primary SG [16]. In our cohort, all three procedures (RYGB, SADI, and BPD-DS) demonstrated a similar rate of early Clavien-Dindo complications and mortality. Reported overall 30-day mortality rates in our large nationwide study were very low, highlighting the safety of revisional bariatric surgery irrespective of procedure choice. Previously, several studies cautioned about the potential higher risks linked to revisional duodenal switch (either SADI or BPD-DS) as well as increased mortality compared to RYGB conversions [17]. However, with advancements and expertise in this technique, more recent studies have demonstrated comparable outcomes in terms of mortality, readmission, reoperation, and reintervention rates between duodenal switch and RYGB [17,18]. It is important to note, however, that we identified higher rates of anastomotic leak and deep surgical site infections rates in our cohort of patients undergoing duodenal switch compared to those undergoing RYGB. These higher rates may be attributed to the longer operative time, higher complexity of the procedure, as well as the extensive surgical manipulation leading to altered gut anatomy and wound healing [17]. Anastomotic leaks can occur up to three weeks after surgery, emphasizing the need for vigilance post-bariatric surgery [19]. Unfortunately, the 30-day limitation of the MBSAQIP fails to fully capture the real-world implications of anastomotic leaks. In fact, some studies showed that leaks are an independent predictor of increased long-term mortality, malnutrition, and overall serious complications [19,20]. This raises the issue of whether the higher incidence of leaks reflects an increase in long-term mortality associated with duodenal switch, a matter that was not covered in this study and requires further long-term evaluation.

Efficient and sustained long-term weight loss after revisional surgery for patients who did not reach optimal weight loss goals after primary SG is a priority. Therefore, in addition to procedure safety, the choice of revisional surgery should also take into account the expected weight loss outcomes. Multiple single-center studies and meta-analyses have compared the weight-loss outcomes between RYGB, SADI, and BPD-DS after initial SG. Jen et al. demonstrated that despite having a statistically higher weight loss at 3 months’ follow-up, revisional RYGB did not show a significantly different excess weight loss (%EWL) in the mid- and long-term period compared to BPD-DS after initial SG [21]. Conversely, other reports describe SADI as having significantly higher short-, mid- and long-term sustained weight-loss results compared to RYGB [22]. These superior outcomes associated with SADI could be explained by the larger section of small bowel bypassed during surgery compared to standard RYGB, which is also associated with higher weight recurrence rates [17]. Given the similar safety profile and higher weight-loss outcomes, SADI emerges as the relatively superior revisional procedure when compared to a standard RYGB. Nevertheless, further long-term studies on this topic are required to aid physicians in procedure selection following primary SG.

In our cohort, longer operative time and Black race were the only independent parameters predicting higher rates of serious complications and mortality after revisional bariatric surgery. Other efforts have been made to identify specific demographical and operative parameters associated with increased rates of complications [23]. In alignment with our results, Inaba et al. highlighted the proportional positive correlation between increased operative duration and mortality after RYGB, among other predictors [24]. Lastly, in an effort to elaborate on the racial disparities in terms of postoperative complications, Nguyen et al. found that Black individuals had 73% higher odds of 30-day mortality using a nationwide inpatient sample in 2013 [25]. Similarly, data from the MBSAQIP in 2015 demonstrated higher reoperation, readmission, and mortality rates for Black patients [26]. Whether these differences are due to socioeconomic factors such as social support, access to transportation, payor status, and physician access or to other ill-defined factors [27,28], these racial disparities shed a light on the need to standardize postoperative treatment and resolve the contributing factors that limit access to care and influence surgical outcomes for specific patients.

### Limitations

This paper is subject to the limitations inherent to any large national database study. These include a lack of granular data, patient selection, and timing between SG and conversion procedures. This lack of granularity may introduce bias. Indications for conversion, which may exist concurrently, are not individually captured by the MBSAQIP. The MBSAQIP only captures the main indication for revision, as secondary indications may include GERD. Given the nature of the MBSAQIP, there was also no data on the sites of the reported leaks. The introduction of new variables may have also been overlooked by the data abstractors. Finally, our outcomes were limited to 30 days, and we were unable to report long-term complications following these surgeries.

## 5. Conclusions

In our cohort, RYGB accounted for the majority of conversions after SG for weight-related indications. Despite SADI and BPD-DS having longer operative times and higher leak rates compared to RYGB, our study demonstrated equal safety in terms of complications and mortality among all three revisional procedures. Further studies are required to evaluate the long-term outcomes of revisional surgery following initial SG.

## Figures and Tables

**Table 1:** Patient baseline characteristics and demographics.

	RYGB	SADI	BPD-DS	*p*-Value
n (%)	n = 5734	n = 642	n = 1012	
Age, years				
mean ± SD	45.0 ± 10.3	44.0 ± 10.2	44.7 ± 10.0	0.116
Female	5019 (87.5)	537 (83.6)	847 (83.7)	<0.001
Race				0.229
White	3189 (55.6)	351 (54.7)	592 (58.5)	
Black or African American	1797 (31.3)	195 (30.4)	303 (29.9)	
Other	748 (13.1)	96 (14.9)	117 (11.6)	
Body mass index (kg/m^2^)				<0.001
mean ± SD	43.2 ± 7.3	45.5 ± 7.8	46.4 ± 8.4	
<40	2109 (85.0)	159 (6.4)	212 (8.6)	
40 to 49	2712 (76.1)	332 (9.3)	521 (14.6)	
50 to 59	754 (70.0)	112 (10.4)	211 (19.6)	
60 to 69	138 (61.6)	36 (16.1)	50 (22.3)	
>70	21 (50.0)	3 (7.1)	18 (42.9)	
Functional status				0.237
Independent	5698 (99.5)	635 (99.8)	1002 (99.1)	
Partially dependent	27 (0.5)	1 (0.2)	9 (0.9)	
Fully dependent	3 (0.0)	0 (0.0)	0 (0.0)	
American Society of Anesthesiologists class				<0.001
1–2	1090 (19.0)	124 (19.3)	147 (14.5)	
3	4486 (78.3)	487 (76.0)	790 (78.1)	
4–5	156 (2.7)	30 (4.7)	74 (7.3)	
Smoker in previous year	273 (4.8)	32 (5.0)	45 (4.5)	0.868
Diabetes				0.015
None or diet-controlled	4986 (87.0)	535 (83.3)	856 (84.6)	
Non-insulin-dependent	584 (10.2)	85 (13.2)	113 (11.2)	
Insulin-dependent	164 (2.9)	22 (3.4)	43 (4.3)	
Hypertension	2155 (37.6)	238 (37.1)	381 (37.7)	0.966
Gastroesophageal reflux disease	2818 (49.2)	234 (36.5)	354 (35.0)	<0.001
Chronic obstructive pulmonary disease	53 (0.9)	5 (0.8)	11 (1.1)	0.807
Hyperlipidemia	1031 (18.0)	116 (18.1)	165 (16.3)	0.427
Chronic steroids	126 (2.2)	17 (2.7)	26 (2.6)	0.625
Chronic kidney disease	15 (0.3)	6 (0.9)	2 (0.2)	0.012
Dialysis-dependent	17 (0.3)	3 (0.5)	1 (0.1)	0.365
History of venous thromboembolism	149 (2.6)	37 (5.8)	39 (3.9)	<0.001
Venous stasis	28 (0.5)	5 (0.8)	3 (0.3)	0.390
Preoperative therapeutic anticoagulant use	155 (2.7)	21 (3.3)	31 (3.1)	0.613
Obstructive sleep apnea	1550 (27.0)	202 (31.5)	322 (31.8)	0.001
History of myocardial infarction	63 (1.1)	6 (0.9)	5 (0.5)	0.202
Previous percutaneous coronary intervention	73 (1.3)	6 (0.9)	11 (1.1)	0.698
Previous major cardiac surgery	44 (0.8)	6 (0.9)	6 (0.6)	0.726

RYGB, conversion from sleeve gastrectomy to Roux-en-Y gastric bypass; SADI, conversion from sleeve gastrectomy to single-anastomosis duodeno-ileal bypass; BPD-DS, conversion from sleeve gastrectomy to biliopancreatic diversion with duodenal switch.

**Table 2:** Perioperative factors and postoperative complications.

	RYGB	SADI	BPD-DS	*p*-Value
n (%)	n = 5734	n = 642	n = 1012	
Operative time, minutes mean ± SD	141.0 (66.1)	149.1 (67.8)	148.8 (63.0)	<0.001
Robotic-assisted	1233 (21.5)	194 (30.2)	353 (34.9)	<0.001
Concurrent paraesophageal hernia repair	1389 (24.2)	98 (15.3)	145 (14.3)	<0.001
Concurrent lysis of adhesions	118 (2.1)	36 (5.6)	8 (0.8)	<0.001
Length of stay, days				
median (interquartile range)	1 (1)	1 (1)	1 (1)	0.008
Anastomotic leak	31 (0.5)	10 (1.6)	13 (1.3)	0.001
Postoperative bleeding	91 (1.6)	6 (0.9)	10 (1.0)	0.177
Reoperation	139 (2.4)	17 (2.7)	27 (2.7)	0.862
Non-operative intervention	112 (2.0)	9 (1.4)	18 (1.8)	0.601
Readmission	365 (6.4)	32 (5.0)	59 (5.8)	0.343
Cardiac events	5 (0.1)	1 (0.2)	0 (0.0)	0.525
Pneumonia	23 (0.4)	1 (0.2)	3 (0.3)	0.575
Unplanned intubation	8 (0.1)	0 (0.0)	2 (0.2)	0.558
Acute kidney injury	6 (0.1)	0 (0.0)	0 (0.0)	0.421
Venous thromboembolism	18 (0.3)	2 (0.3)	4 (0.4)	0.914
Deep surgical site infection	56 (1.0)	10 (1.6)	18 (1.8)	0.049
Wound disruption	5 (0.1)	0 (0.0)	1 (0.1)	0.746
Sepsis	21 (0.4)	0 (0.0)	5 (0.5)	0.236
Cerebrovascular accident	0 (0.0)	1 (0.2)	0 (0.0)	0.005
Serious complications	337 (5.9)	32 (5.0)	61 (6.0)	0.628
Death	6 (0.1)	0 (0.0)	1 (0.1)	0.715

RYGB, conversion from sleeve gastrectomy to Roux-en-Y gastric bypass; SADI, conversion from sleeve gastrectomy to single-anastomosis duodeno-ileal bypass; BPD-DS, conversion from sleeve gastrectomy to biliopancreatic diversion with duodenal switch.

**Table 3:** Significant risk factors for serious complications on multivariable logistic regression.

Risk Factor	Adjusted Odds Ratio	95% Confidence Interval	*p*-Value
Conversion to SADI (vs. RYGB)	0.83	0.57–1.20	0.319
Conversion to BPD-DS (vs. RYGB)	1.02	0.77–1.35	0.898
Female sex	0.96	0.72–1.28	0.773
Longer operative time (per hour)	1.18	1.09–1.28	<0.001
Black race	1.32	1.07–1.28	0.010

RYGB, conversion from sleeve gastrectomy to Roux-en-Y gastric bypass; SADI, conversion from sleeve gastrectomy to single-anastomosis duodeno-ileal bypass; BPD-DS, conversion from sleeve gastrectomy to biliopancreatic diversion with duodenal switch.

## Data Availability

The data presented in this study are available on request from the corresponding author. The data are not publicly available due to confidentiality.
